# Plant Glandular Trichomes as Targets for Breeding or Engineering of Resistance to Herbivores

**DOI:** 10.3390/ijms131217077

**Published:** 2012-12-12

**Authors:** Joris J. Glas, Bernardus C. J. Schimmel, Juan M. Alba, Rocío Escobar-Bravo, Robert C. Schuurink, Merijn R. Kant

**Affiliations:** 1 Department of Population Biology, Institute for Biodiversity and Ecosystem Dynamics, 1098 XH Science Park 904, Amsterdam, The Netherlands; E-Mails: j.j.glas@uva.nl (J.J.G.); b.c.j.schimmel@uva.nl (B.C.J.S.); j.m.albacano@uva.nl (J.M.A.); 2 Department of Plant Breeding, Subtropical and Mediterranean Horticulture Institute “La Mayora” (IHSM), Spanish Council for Scientific Research (CSIC), Experimental Station “La Mayora”, E-29750, Algarrobo-Costa, Málaga, Spain; E-Mail: rocio.escobar@eelm.csic.es; 3 Department of Plant Physiology, Swammerdam Institute of Life Sciences, 1098 XH, Science Park 904, Amsterdam, The Netherlands; E-Mail: r.c.schuurink@uva.nl

**Keywords:** glandular trichome, plant-herbivore interactions, pathogen, Solanaceae, pest resistance, plant defense, leaf hair, tomato, plant breeding, genetic engineering

## Abstract

Glandular trichomes are specialized hairs found on the surface of about 30% of all vascular plants and are responsible for a significant portion of a plant’s secondary chemistry. Glandular trichomes are an important source of essential oils, *i.e.*, natural fragrances or products that can be used by the pharmaceutical industry, although many of these substances have evolved to provide the plant with protection against herbivores and pathogens. The storage compartment of glandular trichomes usually is located on the tip of the hair and is part of the glandular cell, or cells, which are metabolically active. Trichomes and their exudates can be harvested relatively easily, and this has permitted a detailed study of their metabolites, as well as the genes and proteins responsible for them. This knowledge now assists classical breeding programs, as well as targeted genetic engineering, aimed to optimize trichome density and physiology to facilitate customization of essential oil production or to tune biocide activity to enhance crop protection. We will provide an overview of the metabolic diversity found within plant glandular trichomes, with the emphasis on those of the Solanaceae, and of the tools available to manipulate their activities for enhancing the plant’s resistance to pests.

## 1. Introduction

Virtually all plant species possess some kind of hair-like epidermal structures. When these structures are present on the aerial parts of a plant, they are commonly referred to as trichomes, while similar outgrowths from the root are called root hairs. Trichomes—the term deriving from the Greek word “trichos”, which means hair—are, in most cases, not connected to the vascular system of the plant, but instead are extensions of the epidermis from which they originate [1]. Trichomes range in size from a few microns to several centimeters and they exhibit a tremendous species-specific diversity in shape (for examples, see [2]), and, therefore, they are often used as diagnostic characteristics for the identification of plant species, e.g., [3]. Trichomes are mainly found on leaves and stems, but they can also occur, depending on the species, on petals, petioles, peduncles and seeds [1]. Trichomes can be single-celled or multicellular, but the criterion that is mostly used to classify them is whether they are glandular or not [4]. Non-glandular trichomes are present on most angiosperms, but also on some gymnosperms and bryophytes [1]. On the model plant *Arabidopsis*, only non-glandular trichomes can be found, which are unicellular and can be either unbranched, or have two to five branches [5]. These trichomes are polyploid [6] and have been extensively studied with respect to their development, e.g., [7]. In contrast, glandular trichomes are usually multicellular, consisting of differentiated basal, stalk and apical cells and can be found on approximately 30% of all vascular plants [8]. Glandular trichomes have in common the capacity to produce, store and secrete large amounts of different classes of secondary metabolites [8,9]. Many of the specialized metabolites that can be found in glandular trichomes have become commercially important as natural pesticides, but also have found use as food additives or pharmaceuticals [10,11]. For instance, plants of the Lamiaceae, comprising species such as mint (*Mentha x piperita*), basil (*Ocimum basilicum*), lavender (*Lavandula spica*), oregano (*Origanum vulgare*) and thyme (*Thymus vulgaris*), are cultivated for their glandular trichome-produced essential oils [9]. Moreover, artemisinin, a sesquiterpene lactone that is produced in the glandular trichomes of annual wormwood (*Artemisia annua*), is used for the treatment of malaria [12]. In addition, gossypol and related compounds, which are dimeric disesquiterpenes produced by cotton (*Gossypium hirsutum*) trichomes, have strong antifungal activity [13] and are potential natural pesticides [14]. It is for these kinds of specialized metabolic properties, and for the opportunities to modify these properties via genetic engineering, e.g., [15], that trichomes have received increased attention over the past years [16]. By means of this review article, we will provide an introduction into trichome biology, thereby focusing on the biosynthesis and biochemistry of the main trichome-produced compounds, as well as their role in plant resistance. Also, we summarize some approaches that have been undertaken to engineer the metabolism of trichomes, especially those of mint, tobacco (*Nicotiana* spp.) and tomato (*Solanum* spp.).

## 2. Trichome Morphology in Mint, Basil and Tomato

Glandular trichomes can be subdivided in capitate and peltate trichomes. Both types are frequently present in, for example, the Asteraceae, Lamiaceae and Solanaceae. Capitate trichomes typically consist of one basal cell, one to several stalk cells, and one or a few secretory cells at the tip of the stalk [17]. They predominantly produce non-volatile or poorly volatile compounds that are directly exuded onto the surface of the trichome [16]. Peltate trichomes, of which typical examples can be found in mint and basil, consist of a basal cell, one (short) stalk cell, and a head consisting of several secretory cells, which is surmounted by a large sub-cuticular storage cavity. This cavity is formed by separation of the cuticle from the cell wall of the secretory cells [18] and it is filled with the products of the secretory cells, thereby giving these trichomes a characteristic “bulb-like” shape [18]. Cell walls of stalk cells are usually cutinized, presumably to prevent contact of trichome-produced compounds, which can be autotoxic, with other parts of the plant [19].

**Figure 1:**
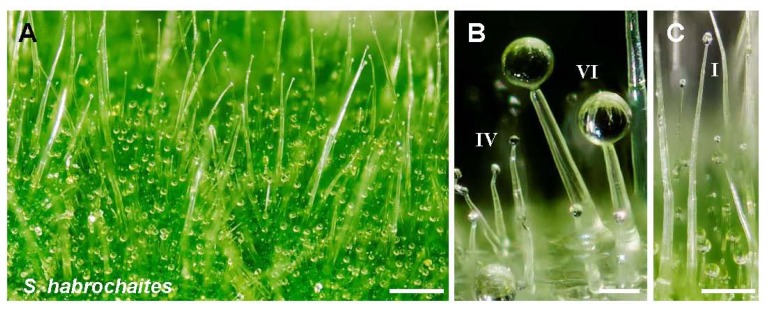

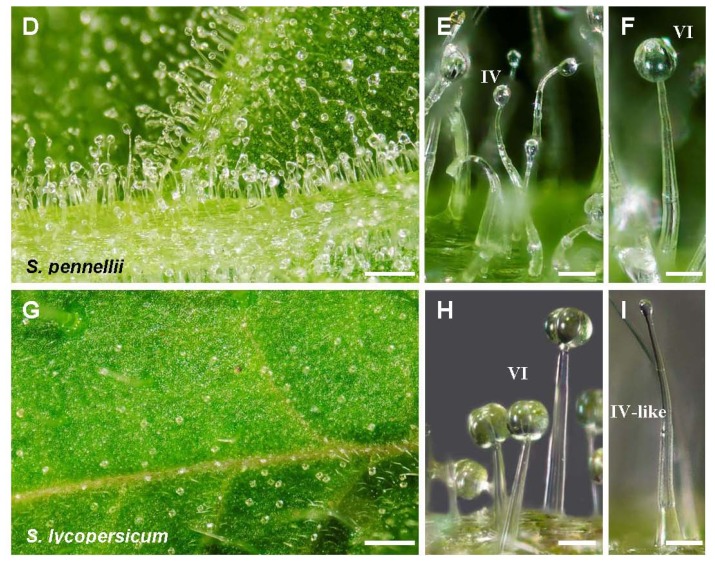
Glandular trichomes in section *Lycopersicon*. Wild accessions have high densities of glandular trichomes that confer resistance to several pests. Panel (**A**) shows the leaflet surface of *Solanum habrochaites* acc. LA 1777 with high densities of glandular trichome types IV and VI (**B**), and type I (**C**). Surface of *Solanum pennellii* acc. LA 716 is also covered by type IV trichomes (**D**, **E**) producing and secreting acyl sugars. This accession also has type VI trichomes, but in low density (**F**). Panel (**G**) shows the surface of *Solanum lycopersicum* cv. Moneymaker. Cultivated tomato has low density of type VI trichomes (**H**) and type I trichomes. Sometimes, type IV-like trichomes (**I**) are observed on stems, veins, and on the leaflet edges. White bars represent 500 μm in panel A, C, D, and G. In panels B, E, F, H, and I, bars represent 50 μm.

The trichomes of the Solanaceae have been studied in detail, especially those of *Solanum* species, because of their role in plant resistance. The morphology of the *Solanum* spp. trichomes was originally described by Luckwill [20], but later revised by Channarayappa *et al.*[21]. Typically, eight different types are distinguished of which four (*i.e.*, type I, IV, VI and VII) are glandular capitate trichomes and four (*i.e.*, type II, III, V and VIII) are non-glandular (Figure 1). Of the glandular trichomes, type I and IV are capitate, whereas type VI and VII are globular. The glandular trichome types differ in number of stalk and secretory cells (see Table 1 for a description of trichome morphology), as well as in their chemical contents.

For example, in the cultivated tomato (*Solanum lycopersicum*), type I trichomes contain mostly acyl glucoses, while type VI trichomes from this species contain terpenoids. Furthermore, the same trichome type can have different content in different tomato species [22]. Trichome type I and IV, which, according to some authors may actually represent the same type, look physically similar to non-glandular trichomes, but they differ by the presence of one or two glandular cells in the tip, which secrete acyl sugars [22]. Type VI glandular trichomes are composed of four secretory cells on a two-celled stalk which secrete metabolites that are stored under a waxy cuticle [22]. In the cultivated tomato, type VI trichomes contain monoterpenes [23,24] as well as a number of sesquiterpenes [24,25]. Interestingly, transcript analysis indicated that both type I and IV, as well as type VI, across *Solanum* species, express many of the genes necessary for acyl sugar, flavonoid and terpenoid production [22]. Type VII glandular trichomes, which are less abundant, consist of a small multicellular glandular head that is situated on a short one-celled stalk [21]. It has been suggested that type VII glandular trichomes of *Solanum habrochaites* are less involved in the biosynthesis of secondary metabolites but instead may have other functions, for instance, protease inhibitor synthesis and storage of alkaloids (*i.e.*, tomatine and dehydrotomatine) and transcripts related to biosynthesis of alkaloids were detected in type VII, but also in type I, IV and VI trichomes of this species [22]. Finally, the presence and density of glandular trichome types differs between *Solanum* species and/or cultivars [21,22,26] (see Table 2 for an overview of trichome morphology across *Solanum* spp.). In addition to the species, trichome density may also depend on the tissue [25] and environmental conditions [27]. Taken together, it is clear that different trichome types have distinct physiological properties and may have evolved due to different selection pressures.

## 3. Biosynthesis and Function of Glandular Trichome-Produced Compounds

The plant epidermal surface represents the first barrier for pathogens and arthropod herbivores [29] to overcome after arrival on a plant. Therefore it may not come as a surprise that trichome density is one of the main factors correlating with resistance to herbivory [26,30]. The presence of trichomes is, however, not always beneficial for the plant, since trichomes may interfere with indirect defense by disturbing natural enemies of herbivores [26,31]. Trichomes can contribute to plant defense in different ways. Non-glandular trichomes can physically obstruct the movements of herbivorous arthropods over the plant surface or prevent herbivores to reach the surface with their mouthparts [32,33]. Moreover, arthropods may become entrapped in sticky and/or toxic exudates, such as acyl sugars or polyphenols, produced by glandular trichomes. Such polyphenols are quickly formed via oxidation when the contents from the glandular trichome heads are released as a result of insect-mediated rupturing of the glandular cuticle. The entrapped herbivores usually die as a result of starvation or of ingested toxins [34] or, in the case of small herbivores, of suffocation [35]. Alternatively, in some cases trichome-produced toxic compounds were found to be transported via the stalk to distal plant tissues, thereby increasing resistance of these tissues against plant attackers, as shown for pyrethrins in the plant pyrethrum (*Tanacetum cinerariifolium*). It appeared that such pyrethrins, produced by glandular trichomes on pyrethrum fruits, can be taken up by the seed and be transmitted to the seedlings, which lack glandular trichomes themselves, resulting in inhibition of fungal growth and of feeding by herbivorous arthropods [36]. Glandular trichomes, thus, function as important chemical barriers for plant parasites [30,37]. The main classes of secondary chemicals that have been found to be produced in trichomes include terpenoids [38], phenylpropenes [39] and flavonoids [40], methyl ketones [41], acyl sugars [42] and defensive proteins [37]. Although all of these compounds play a role in plant defense, both glandular and non-glandular trichomes may have many other functions as well, including attraction of pollinators [4,43], protection against UV due the presence of flavonoids and other UV-absorbing compounds in trichomes [44,45], temperature regulation [43,46] and reduction of water loss [46]. Furthermore, the ability of some plants to tolerate high levels of metals is correlated with their ability to sequester these compounds in their trichomes, as shown for the rough hawkbit (*Leontodon hispidus*) [47], which can sequester calcium, and tobacco (*Nicotiana tabacum*) which is able to secrete cadmium and zinc via its trichomes [48].

### 3.1. Hormonal Regulation of Induced Defenses in Trichomes

In the literature, often two forms of plant defense are discriminated. The first are the constitutive defenses, *i.e.*, those defenses that are always present (such as trichomes), and the second are the induced defenses, which are activated or increased upon attack by herbivores or pathogens (such as some parts of the trichome metabolism). Typically, wounding and/or herbivore infestation activates the octadecanoid pathway, resulting in increasing levels of jasmonic acid (JA) which triggers the expression of defense genes, such as protease inhibitors (PIs), as well as the accumulation of secondary metabolites, like terpenoids [49]. Besides regulating herbivore-induced defense responses, JA is also linked with trichome formation, since JA biosynthesis and reception mutants in the cultivated tomato were shown to have less glandular trichomes [23,50] while, in addition, herbivore feeding as well as JA treatment can give rise to increased trichome densities on newly formed leaves [51–53]. Furthermore, terpene emission can be induced in tomato glandular trichomes by spraying plants with JA [54] and protease inhibitors were shown to be induced in glandular trichomes when trichomes were ruptured by walking insects [50]. Apart from terpenoids [54] and defensive proteins [55], also acyl sugars [55] and alkaloids [56] can be induced in glandular trichomes by spraying plants with MeJA. Thus, JA is essential for induction of defenses in glandular trichomes. Downstream from hormonal regulation, production of many trichome metabolites is also under tight transcriptional control, thereby allowing for temporally regulated emission of, for example, plant volatiles [57,58].

### 3.2. Terpenes

**Figure 2:**
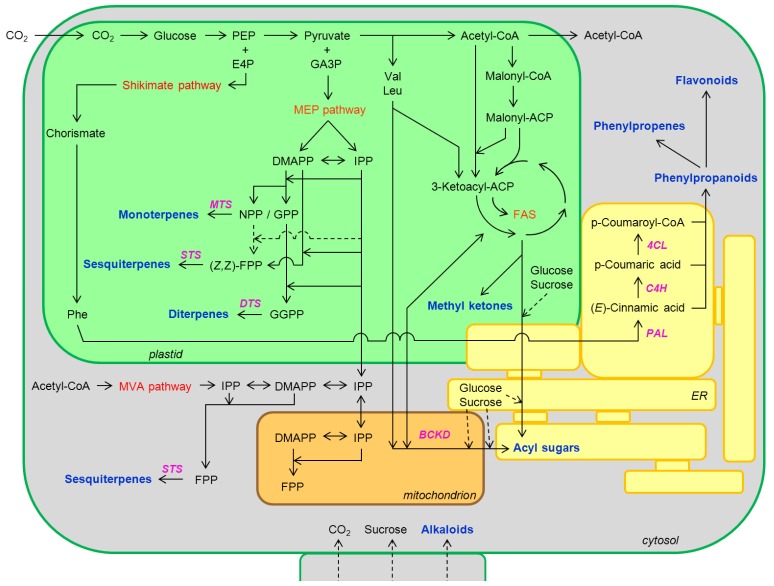
Simplified schematic overview of the biosynthesis of the main secondary metabolites stored and/or secreted by tomato glandular trichome cells. Major pathway names are shown in red, key enzymes or enzyme complexes in purple, and stored and/or secreted compounds in blue. Metabolic routes are projected onto their subcellular location, however final modification reactions (e.g., glycosylations, acylations, methylations, hydroxylations), which can take place at various organelles, are not shown for clarity. Abbreviations used: 4CL, 4-coumarate CoA ligase; ACP, acyl carrier protein; BCKD, branched-chain keto acid dehydrogenase (multi-enzyme complex); C4H, cinnamate 4-hydroxylase; CoA, coenzyme A; DMAPP, dimethylallyl diphosphate; DTS, diterpene synthase; E4P, erythrose 4-phosphate; ER, endoplasmic reticulum; FAS, fatty acid synthesis, FPP, farnesyl diphosphate; GA3P, glyceraldehyde 3-phosphate; GGPP, geranylgeranyldiphosphate; GPP, geranyldiphosphate; IPP, isopentenyl diphosphate; Leu, leucine; the non-mevalonate pathway, also known as the 2-C-methyl-D-erythritol 4-phosphate (MEP) or 1-deoxy-D-xylulose 5-phosphate (DOXP) pathway; MTS, monoterpene synthase; MVA pathway, mevalonate pathway; NPP, neryldiphosphate; PAL, phenylalanine ammonia lyase; PEP, phosphoenolpyruvate; Phe, phenylalanine; STS, sesquiterpene synthase; Val, valine. Solid black arrows indicate established biochemical reactions. Dashed black arrows indicate hypothetical reactions. A single arrow does not necessarily represent a single enzymatic conversion.

With over 30,000 known structures, the terpenoids (or isoprenoids) represent the largest and structurally most diverse class of plant metabolites [59]. Terpenoids play important roles in primary plant metabolism, and provide the building blocks for pigments in photosynthesis (chlorophyll), for electron carriers in respiration (quinone) and for the phytohormones abscisic acid, cytokinins, gibberellins, strigolactones and the brassinosteroids [60,61]. The majority of terpenoids, however, are secondary metabolites and have functions related to plant defense [57]. Despite the immense variety of terpenoids, they are basically all assemblies of C5 isoprene units and produced in three consecutive steps, with a concomitant increase of their complexity and diversity. Since the biosynthesis of terpenoids has been reviewed extensively, we will only highlight the major biosynthetic steps here, for excellent reviews on this topic see e.g., [61–63]. In the cultivated tomato, terpenoids are produced in significant amounts by glandular type VI trichomes [24,25]. The first committed step of terpenoid biosynthesis comprises the formation of the universal C5 “building blocks” isopentenyl diphosphate (IPP) and its isomer dimethylallyl diphosphate (DMAPP). Both IPP and DMAPP are produced via the plastidial 2-*C*-methyl-D-erythritol 4-phosphate (MEP) pathway from pyruvate and glyceraldehyde-3-phosphate (Figure 2) [64,65]. Alternatively, IPP can be formed via the mevalonate (MVA) pathway from acetyl-CoA [66]. It has been suggested that the MVA pathway may partly occur in the peroxisomes, instead of the cytosol, but for tomato, this has not been shown [67]. Subsequent steps of terpenoid biosynthesis may take place at various subcellular locations, for instance, in the plastids, the (smooth) endoplasmic reticulum, mitochondria and/or the cytoplasm and, in line with this, different isoforms of the enzyme isopentenyl diphosphate isomerase (IDI), which catalyzes the isomerisation of IPP to DMAPP, can be found in the plastids, mitochondria and/or cytosol [68–70]. Furthermore, IPP and other terpenoid intermediates can also be shuttled between organelles [61,69]. Evidence for transport of DMAPP to other cellular compartments is lacking, or perhaps DMAPP is not transported at all [69]. In tobacco, the presence of chloroplasts in trichomes was shown to be necessary for production of diterpenes [71], thereby confirming the importance of these organelles in terpenoid biosynthesis.

In the second step of terpenoid biosynthesis, a single (C5) DMAPP serves as the substrate for successive head-to-tail condensations of one or more C5 IPP units. These linear chain elongation reactions are catalyzed by homo and/or heteromeric complexes of prenyltransferases [72]. Any of the intermediate products can be used as starting material for the synthesis of short (up to C20) isoprenyl diphosphates [61,73]. Interestingly, while most isoprenyl diphosphates are generated only in the cis (*Z*) or trans (*E*) conformation, some are produced in both isoforms [24,74]. The head-to-tail condensation reactions lead to the formation of C10 (*E*)-geranyl diphosphate (GPP) and (*Z*)-neryl diphosphate (NPP), the C15 (*E*,*E*)-farnesyl diphosphate (FPP) and (*Z*,*Z*)-farnesyl diphosphate (*Z*,*Z*-FPP), the C20 (*E*,*E*,*E*)-geranylgeranyl diphosphate (GGPP) (Figure 2), and the longer oligoprenyl diphosphate (OPP; C25-45) and polyprenyl (C50-130) terpenoid precursor molecules. In the final step, the (*Z*)- or (*E*)-isoprenyl diphosphates are converted into cyclic and acyclic terpenoids, catalyzed by a large enzyme family of terpene synthases (TPSs) [75,76]. The newly formed terpenoids are often subject to (multistep) secondary transformations, catalyzed by various enzymes in different organelles [62,77], leading to a wide range of structurally related terpenoids, which can be non-volatile like pigments and phytohormones, or volatile like the hemiterpenes (C5; derived from DMAPP), monoterpenes (C10), sesquiterpenes (C15), diterpenes (C20), triterpenes (C30), *etc.*, and norterpenes (e.g., C11 and C16) [61,63,78]. Most terpene synthases are able to generate multiple products from a single substrate, which, together with the large size of TPS gene families, explains the diversity of terpenoids found in plants [62,77].

Terpenoids are major components of herbivore-induced volatile blends and they play an important role in the attraction of predators and parasitoids to herbivore-infested plants, a phenomenon known as indirect plant defense [79,80]. Indirect defenses mediated by plant volatiles have been reported from plant species with glandular trichomes, including model plants like cultivated tobacco [81], corn (*Zea mays*) [80], cotton [81] and cultivated tomato [49], but also from species without glandular trichomes, for example Arabidopsis (*Arabidopsis thaliana*) [82] and lima bean (*Phaseolus lunatus*) [83]. Terpenes may also play a role in direct defenses against pests as they can have a deterrent or repellent effect and at higher concentrations they are often toxic. For instance, in the wild potato (*Solanum berthaultii*), the release of the sesquiterpene (E)-β-farnesene from its glandular trichomes was shown to repel aphids (*Myzus persicae*) [84], while the parasitoids of this aphid, like the hymenopteran *Diaeretiella rapae*, were attracted to (E)-β-farnesene [85]. The sesquiterpenes 7-epizingiberene and *R*-curcumene, produced by glandular type VI trichomes of some *Solanum* species [30], were shown to have a repellent effect on silverleaf whiteflies (*Bemisia tabaci*) [86,87]. Other herbivorous arthropods are affected as well by sesquiterpenes like zingiberene. For example, Carter *et al.*[88] showed that zingiberene is toxic to Colorado potato beetle (*Leptinotarsa decemlineata*) larvae and removal of sesquiterpenes by wiping *S. habrochaites* foliage with methanol increased the survival of beet armyworm larvae (*Spodoptera exigua*) from 0% to 65% [89]. In the South American tomato pinworm (*Tuta absoluta*), the presence of zingiberene was associated with a reduction in oviposition and feeding damage [90]. Finally, increased zingiberene levels were shown to correlate with increased repellency of the tobacco spider mite (*Tetranychus evansi*) [91].

### 3.3. Phenylpropenes

Like terpenoids, phenylpropanoids exhibit great structural diversity [92] and are emitted in significant amounts by plants, but both the quantity and the composition of the phenylpropanoid blend can markedly differ between species [93] and even cultivars [94]. Despite this structural diversity, three successive, very conserved, enzymatic conversions form the core of the phenylpropanoid biosynthetic pathway (Figure 2) [92]. The first committed step comprises the non-oxidative deamination of phenylalanine to trans-cinnamic acid, catalyzed by phenylalanine ammonia lyase (*PAL*). Next, trans-cinnamic acid is hydroxylated to para-coumaric acid by cinnamate 4-hydroxylase (*C4H*). Finally, para-coumaric acid is activated by 4-coumarate CoA ligase (*4CL*), creating para-coumaroyl CoA, which is the general precursor for a wide range of products, including anthocyanins, flavonoids, lignin and phenylpropenes [57,92]. Together with terpenoids, the phenylpropenes are the major constituent of essential oils, which are secreted from glandular trichomes of many Lamiaceae [62]. In basil, for instance, eugenol and methylchavicol were shown to be predominantly synthesized and stored in the glandular trichomes [39].

Benzenoids, which are derived from trans-cinnamic acid by shortening of the side-chain [95,96], do not appear to be emitted from foliar glandular trichomes in large amounts and/or by many plant species. For instance, van Schie *et al.*[54] did not find evidence for production of methyl salicylate in tomato glandular trichomes and glandular trichomes of alfalfa (*Medicago sativa*) and hop (*Humulus lupulus*) emit only small amounts of benzenoids [97]. In contrast, methyl cinnamate, which is produced by methylation of trans-cinnamic acid, is synthesized in significant amounts by glandular trichomes [98].

Compared to the extensive knowledge on terpenoid biosynthesis, relatively little is known about the biosynthesis of eugenol, chavicol and their derivatives. The intermediate steps that follow after coumaric acid has been synthesized remain unclear, although an enzyme was identified in basil glandular trichomes that could catalyze the formation of eugenol by using coniferyl acetate and NADPH as substrates [99]. Furthermore, *O*-methyltransferases responsible for the last step in the formation of methylchavicol and methyleugenol have been characterized and were highly expressed in basil glandular trichomes [100].

Phenylpropenes are well known for their role in the attraction of pollinators. For example, methyleugenol from the orchid *Bulbophyllum cheiri* was shown to attract several fruit fly species (Bactrocera spp.) for pollination [101]. Furthermore, although the evidence is limited, some studies suggest that eugenol may contribute to plant resistance by negatively affecting plant parasites. For example, application of synthetic eugenol caused mortality and repellency in 4 Coleopteran species [102]. Moreover, also nematodes appeared to be susceptible to eugenol [103], as well as some fungi such as *Cladosporium herbarum* in which eugenol caused morphological deformations of the hyphae [104]. Taken together, it is clear that phenylpropenes fulfill dual roles, both in defense against herbivores, as well as in attraction of pollinators.

### 3.4. Flavonoids

Like the phenylpropenes, flavonoids are derivatives from the phenylpropanoid pathway. The first step in flavonoid biosynthesis comprises the condensation of one molecule of 4-coumaroyl-CoA and three molecules of malonyl-CoA, catalyzed by the enzyme chalcone synthase (CHS), followed by a cyclization reaction. In subsequent reactions, the flavone basic structure can be further modified by reductases, isomerases, hydroxylases, and glycosyltransferases, thereby forming the various subclasses of flavonoids, such as flavones, flavonols, flavandiols, anthocyanins, proanthocyanidins and isoflavonoids [105]. Accumulation of flavonoids in trichomes may serve to protect plants from UV-B [45] and there is evidence for sunlight-induced secretion of flavonoid glycosides by glandular trichomes of *Phillyrea latifolia* plants to protect them against damage induced by UV-A [106]. In *S. habrochaites*, it was shown that type I, IV and VI glandular trichomes contain methylated forms of the flavonol myricetin [107]. In the cultivated tomato, it was subsequently shown that the *hairless* (*hl*) mutation, which causes alterations in the morphology of all trichome types, also decreased accumulation of quercetin-trisaccharide, rutin, kaempferol-rhamnoside and 3-*O*-methylmyricetin in type VI glandular trichomes [25,108]. These and related phenolic compounds can inhibit growth of lepidopteran larvae [109]. Interestingly, trichomes from *hl* leaves were also deficient in various sesquiterpenes, but contained wt levels of monoterpenes and acyl sugars [25]. As suggested by Kang *et al.*[25], perhaps *hl* disrupts a cellular function required for the biosynthesis of sesquiterpenes and flavonoids, which are both synthesized in the cytosol.

### 3.5. Methyl Ketones

Methyl ketones constitute a class of fatty-acid derived volatile compounds that are very effective in protecting plants against pests [30]. Methyl ketones that are commonly found in plants have 7 to 15 carbons and include 2-heptanone, 2-nonanone, 2-undecanone, 2-tridecanone and 2-pentadecanone [41]. In *S. habrochaites*, methyl ketone biosynthesis was shown to proceed in two steps. The first step comprises the hydrolysis of 3-ketoacyl-acyl carrier protein intermediates, produced during fatty acid biosynthesis in chloroplasts (Figure 2). This step is catalyzed by an enzyme identified as methyl ketone synthase 2 (MKS2) [110,111]. The resulting 3-ketoacids are then decarboxylated in a reaction that is catalyzed by MKS1 [41,111].

In the 1980s, 2-tridecanone was identified as the major constituent of type VI trichomes of the wild tomato *S. habrochaites* f. *glabratum*[112]. Methyl ketones in this species were found in concentrations between 2700 and 5500 μg per g fresh weight, whereas the cultivated tomato also contains 2-tridecanone, but in much smaller amounts, of up to 80 μg per g fresh weight [113]. Williams *et al.*[112] demonstrated that 2-tridecanone was lethal to several herbivorous arthropods, including the tobacco hornworm (*Manduca sexta*) and the cotton aphid (*Aphis gossypii*). Tomato fruitworm (*Helicoverpa zea*) larvae were shown to be killed by the fume of *S. habrochaites* f. *glabratum* and by pure 2-tridecanone [114]. Chatzivasileiadis *et al.*[115] showed that methyl ketones are toxic to the two-spotted spider mite upon contact. Trichome exudates and 2-tridecanone applied on artificial membranes inhibited feeding and caused mortality of the potato aphid (*Macrosiphum euphorbiae*) [116]. A second methyl ketone from tomato, identified as 2-undecanone [117], appeared to be less toxic since it did not negatively affect the potato aphid [116] nor did it cause larval mortality in the tobacco hornworm [117]. 2-undecanone did, however, cause increased mortality in the two-spotted spider mite [115] and it also increased mortality of pupae of the tomato fruitworm, and even more so when larvae of this species were reared on an artificial diet containing both 2-tridecanone and 2-undecanone [117].

### 3.6. Acyl Sugars

Sugar esters, also called acyl sugars, are nonvolatile metabolites, produced [118] and stored in glandular trichomes of many Solanaceae, including *Solanum, Nicotiana*, *Datura*[42] and *Petunia* species [119]. These compounds are conjugates of sugars and aromatic or aliphatic fatty acids and a significant fraction of these are exuded onto the surface of aerial organs, in the case of the wild tomato *Solanum pennellii* up to 20% of the plant’s leaf dry weight [120]. Acyl sugar biosynthesis is especially well studied in tomato [118,121] and tobacco [118,122] species. The backbone of acyl sugars consist of a sugar, predominantly sucrose or glucose, or sometimes a sugar-alcohol, predominantly sorbitol of xylitol, to which one or more straight or branched chain fatty acids, which are usually methyl-branched, are esterified. Depending on the number of acyl groups, *i.e.*, the free hydroxyl groups in the sugar, most of these sugar esters are mono-, di- or tri-acyl sugars [35] and are formed via *O*-acylation. For example, type IV glandular trichomes of *S. pennellii* exude a mixture of 2,3,4-*O*-tri-acyl-glucoses [123], 3′,3,4-*O*-tri-acyl-sucrose and 3′,3,4,6-*O*-tetra-acyl-sucrose polyesters, which have both straight and branched chains, ranging in length from 2 to 12 carbons, that are formed prior to acetylation to glucose and sucrose [9,124,125]. The branched- or straight-chained fatty acid acyl moieties of the glucose esters of *S. pennellii* are derived from branched-chain amino acids (*i.e.*, Val, Leu, and Ile) [124]. In *Solanum* and *Datura* species, elongation of fatty acids is mediated via fatty acid synthase (FAS), while in tobacco and petunia this elongation occurs via α-ketoacid elongation [42]. Biosynthesis and elongation of branched fatty acids involves the branched-chain keto acid dehydrogenase (BCKD) protein complex which generates activated acyl-CoA esters from branched-chain keto acid precursors [118] but how these acyl-CoA esters are exactly used for synthesis of acyl sugars is still unclear [121]. In *S. pennellii*, the acylation steps require sequential action of a glucosyl transferase, which forms the first acyl sugar intermediate, and an acyl transferase that catalyzes the further additions of fatty acids to the backbone [126,127]. Finally, also an acyltransferase (*AT2*) has been identified that catalyzes the transfer of the acetyl group found in the tetra-acyl sucroses of the cultivated tomato [121]. Expression of *AT2* was shown to be specific for the tip cells of type IV glandular trichomes of an *S. lycopersicum x S. penelli* introgression line [121].

Acyl sugars may be directly toxic to herbivores, but they are also excellent emulsifiers and surfactants and may easily stick to arthropod cuticles thereby immobilizing or suffocating arthropods [1,35]. Wagner *et al.*[1] reported that aphids upon contact with tobacco trichomes are rapidly “coated” by trichome-produced sugar esters, thereby entrapping the insect and preventing it from further moving around. Staining with Rhodamine B revealed that the highest concentrations of sugar esters are present at the joints of the aphid’s antennae and legs where entry of toxins into the body is likely to occur most easily [1]. Also, it was shown that acyl sugars can deter or repel herbivores, such as the potato aphid. Structure and activity studies revealed that acyl glucoses and acyl sucroses were equally repellent to the aphid and differences in the length of the fatty acid chain did not influence repellency [128]. However, according to Puterka *et al.*[35] the toxic properties of synthetic acyl sugars depend both on sugar backbone and fatty acid chain length, and different acyl sugars caused different mortalities in pear psyllids (*Cacopsylla pyricola*), tobacco aphids (*Myzus nicotianae*), tobacco hornworms and spider mites. Furthermore, in tomato the density of glandular trichomes and the amount of acyl sugars were shown to correlate with resistance to whiteflies and spider mites [129–131]. Other arthropods that were shown to be negatively affected by acyl sugars include the tomato fruitworm, the beet armyworm (*Spodoptera exigua*) [132] and the leafminer (*Liriomyza trifollii*) [133]. Apart from functioning as direct defense, acyl sugars may also function in indirect defenses. Although perhaps counter-intuitive, it appeared that freshly hatched larvae of three Lepidopteran herbivore species, *i.e.*, the beet armyworm, the tobacco hornworm and the African cotton leafworm (*Spodoptera littoralis*), preferred to feed from trichomes as their first meal and were not negatively affected by this. However, it was found that this behavior could backfire depending on the ecological setting of the animals, as the high concentration of ingested and digested acyl sugars caused these larvae to release a distinct odor of branched-chain fatty acids from their body and frass. This odor appeared sufficient to betray their whereabouts to one of their natural enemies, the omnivorous ant *Pogonomyrmex rugosus*[122].

### 3.7. Defensive Proteins

Apart from secondary metabolites, trichomes are also able to produce significant amounts of proteins with defensive functions, such as proteinase inhibitors (PIs) [134], polyphenol oxidases (PPOs) [135] and phylloplanins [37]. PIs can be either constitutively expressed (e.g., in flowers) or induced upon wounding or herbivory in leaves and their trichomes [53] and induced PIs slow down the growth of herbivores upon ingestion [136,137] probably via inhibition of digestive proteinases in the herbivore gut. PPOs constitute a class of enzymes that utilize molecular oxygen for the oxidation of mono- and *O*-diphenols to *O*-dihydroxyquinones [138]. Significant amounts of PPOs can accumulate in trichomes. For instance, in glandular trichomes of the wild potato, PPO can constitute up to 70% of the total protein content [139]. In the cultivated tomato, there is evidence that some isoforms of the PPO family are expressed in specific trichome types and not in others [140]. For example, PPO-A and C are expressed in type I and IV trichomes, as well as PPO-E and F while, in contrast, type VI trichomes express PPO-D, E and F, but not A and C. PPOs and their substrates are compartmentalized probably to prevent spontaneous reactions. In the head cells of tomato type VI trichomes, PPOs are stored in leucoplasts whereas their phenolic substrates are present in the vacuoles [140]. When the tissue is damaged, for instance by walking herbivores, the PPOs will mix with vacuolar content of the head cell and rapidly oxidize *o*-dihydroxyphenolics to the corresponding *O*-quinones [141]. These quinones, in turn, are highly reactive molecules that covalently bind to nucleophilic -NH2 and -SH groups of molecules such as amino acids and proteins, thereby reducing the availability of essential amino acids to the herbivores and/or the digestibility of proteins [141,142], or perhaps interfering directly with enzymes. Apart from reducing the nutritive quality of leaves to herbivores [141], trichome-PPOs have also been implicated in resistance to plant pathogenic bacteria. Overexpression of a PPO from potato (*Solanum tuberosum*) in cultivated tomato yielded transgenic plants that were much more resistant to the bacterial pathogen *Pseudomonas syringae*[143,144], and in dandelion (*Taraxacum officinale*) suppression of *PPO-2* via silencing increased plant susceptibility to *P. syringae*[145]. Glandular trichomes may also actively secrete proteins, as shown in cultivated tobacco, where proteins can be deposited on the leaf surface through pores that are present in the cuticle of short glandular trichomes [37,146] which are reminiscent of tomato type VII trichomes [147]. These secreted proteins, termed tobacco phylloplanins, inhibited spore germination and leaf infection by the oomycete pathogen *Peronospora tabacina*[37]. It has been suggested that these proteins, possibly in interaction with other secreted trichome-produced compounds, are broadly distributed over the leaf surface of tobacco plants, thereby providing constitutive resistance against diseases [148].

## 4. Identification of Biochemical Pathways in Glandular Trichomes

Interest in trichome-produced compounds, combined with technical breakthroughs in analytical equipment and the possibility of applying genomic approaches, has greatly increased the understanding of the biochemical pathways that operate in trichomes, as well as the products they generate. Sequencing of Expressed Sequence Tag (EST) libraries generated from mRNA from isolated trichomes has resulted in large databases, which, in combination with metabolite profile analysis of glandular trichomes and proteomics, has led to a much more detailed general insight into the biosynthesis of these specialized metabolites than obtained previously via chemical-analytical methods [16].

Based on analyses of EST databases, it has been suggested that trichomes operate mostly as a self-supporting system [9] and have highly active biochemical pathways for both primary and secondary metabolism [9]. In glandular trichomes of tomato and tobacco, genes encoding enzymes and proteins related to photosynthesis and carbon fixation are significantly expressed [149,150], indicating that at least some of the carbon necessary for secondary metabolism can be fixed within trichome cells [22]. Earlier studies in cultivated tobacco had also indicated that glandular trichomes possibly can fix carbon and produce sugar and diterpenoids (*i.e.*, duvatrienediol) independent from the rest of the plant, although a role for additional carbon imported from the tissues below the trichome cannot be excluded [151,152]. In mint, however, photosynthesis-related genes were not expressed [153], indicating significant differences between plant species or between trichome types. It has been suggested that the total amount of secondary metabolites produced by glandular cells could be related to their capacity to fix carbon [154] since exudates from photosynthetically active glandular cells can constitute up to 20% of the leaf dry weight biomass in wild Solanaceae [118,120] while exudates from species with leucoplasts instead of chloroplasts in their trichomes, like the Lamiaceae and Fabaceae, contribute less than 2% to the leaf dry weight [39,62,154,155]. Leucoplasts are non-pigmented plastid-type organelles specialized for *de novo* biosynthesis of (precursors for) the metabolites that often will be secreted [18]. However, photosynthetically active glandular trichomes are probably supplemented with carbon substrates (e.g., CO_2_, sucrose, glucose) as well, because their own primary metabolism is most likely incapable of meeting the huge carbon demands [22,154]. Besides carbon, other compounds, like nitrogen, phosphate and micronutrients, are required for metabolism as well. According to Schilmiller *et al.*[9], import of amino acids into trichomes is minimal, begging the question of how trichomes acquire their nitrogen necessary for the large amount of proteins synthesized in trichomes [37,139]. Possibly, nitrogen can be recycled by amino transferases in glandular trichomes [9], but it cannot be excluded that nitrogen and other essential substances are imported via the trichome stalk, as well.

EST analyses have played an important role in identifying enzymes of trichome secondary metabolism, for instance, in the synthesis of geraniol [156] and eugenol [99] in basil; the synthesis of methyl ketones [41,110], monoterpenes [24] and sesquiterpenes [74,157,158] in *Solanum* species; and, xanthohumol synthesis in hops [159]. In mint, the first species from which trichome-specific ESTs were sequenced, 35% of the sequences were estimated to be involved in secondary metabolism of which 25% in monoterpene biosynthesis [153]. Genes from primary metabolism pathways were found to be highly expressed in mint trichomes, with, for example, genes of the glycolytic pathway, the pentose phosphate and the oxidative phosphorylation pathway accounting for more than 35% of all ESTs as well as lipid transfer protein (LTP) homologs (32%), which probably play a role in metabolite transport [16,153] since, in tobacco, the trichome-specific *LTP1* gene was shown to play a role in the secretion of terpenoids [160]. In contrast, in basil, more than 25% of glandular trichome ESTs were related to the phenylpropanoid pathway or involved in phenylpropene biosynthesis [39]. In basil, tobacco and cultivated tomato, proteomics studies on glandular trichomes have been carried out as well, allowing for a detailed comparison between their transcriptomes and proteomes [e.g., 157,161] and this led to new insights in the posttranscriptional regulation of trichome metabolism [161]. Included among the many proteins (1552 in total) identified in tomato trichomes were: enzymes involved in the MEP pathway; enzymes involved in synthesis of the flavonoid compound rutin; enzymes that take part in synthesis of volatile aldehydes (e.g., lipoxygenase C and hydroperoxide lyase; HPL); and, defense-related proteins, such as PPOs [157]. Moreover, a sesquiterpene synthase was identified that produces β-caryophyllene and α-humulene from *E,E*-farnesyl diphosphate in glandular trichomes of leaves, but not in glandular trichomes of the stem [157], while other sesquiterpene synthases are preferentially expressed in other organs, for instance, in glandular trichomes of the stem [76,158]. This indicates that, depending on the plant organ, there can be differences between if and when genes are expressed in glandular trichomes and thus which metabolites they accumulate.

## 5. Trichome Engineering to Increase Plant Resistance

The preparation of trichome-specific EST databases did not only facilitate the discovery and characterization of genes in trichome biosynthetic pathways, but also made it more feasible to engineer the production of specific biocides in trichomes [147]. Most engineering strategies are not designed to obtain expression of a transgene exclusively in a target tissue, such as a trichome, and/or at specific moments, but make use of a construct in which the transgene is fused behind the general 35S promoter and is expressed either via stable transformation [15] or via virus-induced gene silencing [121,162]. Although this can give rise to pleiotropisms [163], such strategies clearly can suffice to manipulate key metabolic steps in the target biocide’s metabolic route. Cultivated tobacco, and its related wild species *Nicotiana sylvestris* and *Nicotiana tomentosiformis*, produce diterpenes, exist in two forms: the macrocyclic cembranoids, including the cembratrien-diols (CBT-diols) and their precursors the cembratrien-ols (CBT-ols), and the bicyclic labdanoids. Cultivated tobacco and *N. tomentosiformis* produce both labdanoids and cembranoids, while *N. sylvestris* only produces the latter group of compounds. These diterpenes are produced in large amounts and, specifically, in the glandular capitate trichomes of the plant, and some of these labdanoids contribute to plant resistance to pests, making tobacco an ideal target for terpenoid metabolic engineering [147]. For example, in cultivated tobacco, downregulation of a trichome-specific CYP450, a CBT-ol hydroxylase, via antisense suppression, led to reduction of CBT-diol levels but promoted the levels of its insecticidal precursor CBT-ol, thereby increasing plant resistance to the red aphid (*Myzus nicotianae*) [164]. Thus, silencing genes can be used to increase levels of compounds with biocidal properties in trichomes, thereby enhancing a plant’s resistance. The large amount of data collected on trichome-specific gene expression [16,157,158] also made it possible to tailor gene overexpression more specifically by using trichome-specific promoters instead of the 35S promoter. The promoter of the trichome-specific CBT-ol hydroxylase gene [164] was one of the first trichome-specific promoters that has been isolated [165] and in *N. sylvestris* several *cis*-regulatory elements of a CBT-ol synthase promoter were identified, required for specific expression in the secretory cells of glandular trichomes [166] and the CBT-ol synthase promoters have been used to produce novel diterpenoids [167] and heterologous sesquiterpenes in *N. sylvestris*[74]. Moreover, from squash (*Cucurbita maxima*) Anandan *et al.*[168] isolated the promoters of a protease inhibitor family and found that one of these was trichome-specific while Liu *et al.*[169] cloned the cotton fiber-specific *LTP3* promoter and fused it to ß-glucuronidase (GUS), and demonstrated that expression of this construct in transgenic tobacco plants indeed was specific for its trichomes. However, the possibilities to modify plant–pest interactions by altering trichome chemistry via herbivore- or pathogen-specific promoters have hardly been addressed. Van Schie *et al.*[54] characterized a trichome-specific linalool synthase, called MTS1, induced by wounding the plant defense-hormone JA and by spider mite feeding and, potentially, the promoters of such herbivore-inducible trichome-specific genes can be used to re-engineer trichome based resistance. Finally, Bleeker *et al.*[170] provided proof of this concept by demonstrating that expressing 7-epizingiberene synthase from *S. habrochaites* fused to the MTS1 promoter [54], together with *Z*-*Z*-farnesyl-diphosphate synthase fused to the MKS1 promoter [111], specifically in the glandular trichomes of the cultivated tomato can improve resistance against herbivores, including whiteflies and spider mites.

In conclusion, glandular trichomes are an important first line of defense against herbivorous insects and pathogens. Tremendous progress in the availability of genomic data has allowed for the discovery of genes in various biosynthetic pathways involved in trichome-produced compounds. However, the full potential of trichomes has not been exploited even remotely since plant secondary metabolism is complex and multilayered while our knowledge on the precise actions of the different members of large gene families and on the rate-limiting steps in pathways is still too incomplete to make the outcome of such manipulations easily predictable. However, it is evident that via breeding or genetic engineering—by using, for example, trichome-specific promoters—we will develop a stronger grip on how to obtain the desired levels of biocides in a tissue-specific manner. Thus, these minute glandular trichomes may soon prove to be the ideal vehicles for targeted modification of the versatile secondary metabolism of many plant species to customize essential oil production and enhance biocide-based protection of crops.

## Figures and Tables

**Table 1:** Trichome description according to Luckwill [20] and revised by Channarayappa *et al.*[21].

Type	Description
I 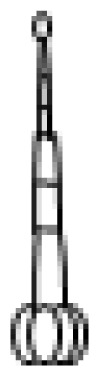	Thin glandular trichomes consisting of 6–10 cells and 2–3 mm long. Globular and multicellular base with a small and round glandular cell in the trichome tip.
II 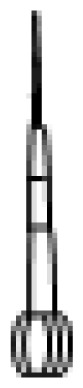	Similar to trichome I but non-glandular and shorter (0.2–1.0 mm). Globular and multicellular base.
III 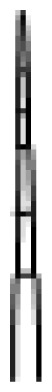	Thin non-glandular trichome consisting of 4–8 cells and 0.4–1.0 mm long with a unicellular and flat base. External walls lack intercellular sections.
IV 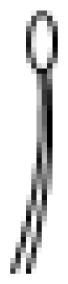	Similar to trichome I but shorter (0.2–0.4 mm) and with a glandular cell in the tip. Trichome base is unicellular and flat.
V 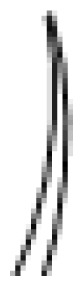	Very similar to type IV with respect to height and thickness but non-glandular.
VI 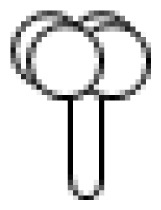	Thick and short glandular trichomes composed of two stalk cells and a head made up of 4 secretory cells.
VII 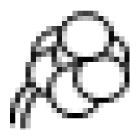	Very small glandular trichomes (0.05 mm) with a head consisting of 4–8 cells.
VIII 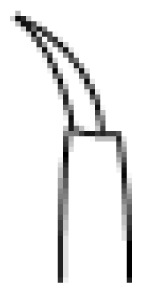	Non-glandular trichome composed of one basal and thick cell with a leaning cell in the tip.

**Table 2:** Distribution of trichome types in the section *Lycopersicon* of the genus *Solanum*.

Species	I 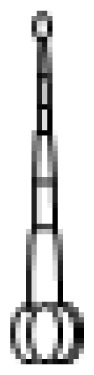	II 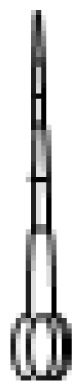	III 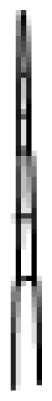	IV 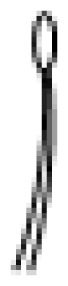	V 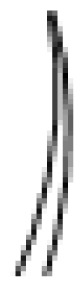	VI 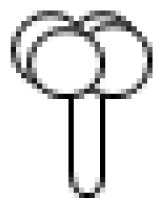	VII 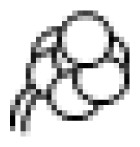	VIII 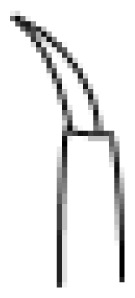
*S. habrochaites*	+		+	+		+	+	
*S. lycopersicum*	+		+		+	+	+	+
*S. pennellii*				+		+		
*S. cheesmaniae, S. galapagense*					+			
*S. pimpinellifolium*		+		+ b	+	+		
*S. peruvianum, S. arcanum, S. corneliomuelleri, S. huylasense*	+	+ a			+	+	+	
*S. chilense*					+	+		+
*S. chmielewski*					+	+		
*S. neorickii*					+	+		

a Described in the form *glandulosum*[20], formally *S. corneliomuelleri*;

b Described in the accession TO-937 [28].
